# Timed image naming evaluation for adults (TIME) using BOSS images

**DOI:** 10.1371/journal.pone.0341774

**Published:** 2026-03-09

**Authors:** Sabine Heuer, Corey Briska, Priyanka Shah-Basak, Sara Pillay

**Affiliations:** 1 Department of Communication Sciences and Disorders, University of Wisconsin-Milwaukee, Milwaukee, Wisconsin, United States of America; 2 Department of Neurology, Medical College of Wisconsin, Milwaukee, Wisconsin, United States of America; University of Bologna, ITALY

## Abstract

Normative data for naming photographs are essential in psycholinguistic research. However, image naming norms are typically derived from young adults, limiting their relevance for older populations, who are at greater risk for language impairments due to neurological conditions such as stroke, traumatic brain injury or dementia. Further, lexical retrieval declines also in healthy aging, making it essential to establish norms for older adults to distinguish normal from impaired word retrieval. This study provides normative data for 600 photographs of the Bank of Standardized Stimuli (BOSS) focusing on three age cohorts (40–50, 51–65, and 66+). We examined naming accuracy, name agreement, H values, and response times (RT) to explore age-related differences in image naming. Participants completed a web-based oral picture naming task via video conferencing. Results revealed overall high naming accuracy (mean = 80.5%) and name agreement (mean = 87.4%) across the full sample, with modest variability across the range of adults self-reportedly free of neurological deficits. The 51–65 cohort showed the highest accuracy and fastest RTs. Significant correlations between RT and name agreement and H value support the inclusion of RT as key indices of naming difficulty. We discuss the implications of these findings considering psycholinguistic norms, demographic influences, and methodological differences from previous image norming studies. Novel contributions of this study include normative data for a large sample of middle to older age adults including RT and alternative names, expanding the utility of the BOSS image set for examining aging-related changes in lexical access. The study underscores the importance of including RT measures alongside traditional naming norms for improved characterization of visual stimuli. Open access to the updated dataset aims to facilitate future research into age-related language processing and supports personalized applications in cognitive and clinical settings.

## Introduction

Image naming tasks are a cornerstone of psycholinguistic, clinical, and educational research, providing a direct window into the cognitive-linguistic processes that support word retrieval. These tasks often involve naming everyday objects, with performance providing insights into lexical processing. To minimize confounding factors, researchers increasingly rely on standardized image norms that control for physical and psycholinguistic variables [1,2]. In this study, we collected image norms from 82 self-reportedly healthy individuals aged 40 years or older, including norms on naming accuracy and response times (RT) using a large set of photographs from the Bank of Standardized Stimuli (BOSS; 1,3). The dataset was originally created for a speech therapy intervention project but here is used to establish normative data for middle to older adults, a population largely underrepresented in existing imaging norming studies.

Image norming typically involves participants evaluating images and providing names, from which indices such as name agreement and image agreement are computed [1,3]. Many norming studies also collect subjective Likert-scale ratings for familiarity, visual complexity, or imageability. Table 1 provides definitions and descriptions of these key computational norms.

**Table 1:** Computational Norms for Image Naming.

Term	Definition
Modal Name	The name provided by the majority of individuals for a given image
H Value	A measure of name agreement variability (Lachman, 1973). A low H value (close to 0) indicates high name agreement, while a higher value indicates greater variability in naming.
Name Agreement	The proportion of individuals providing the same name for an image; high name agreement predicts faster and more accurate naming.
Naming Accuracy	The proportion of individuals providing an acceptable response, including alternate correct names (e.g., “couch” for “sofa”).
Naming Response Time	The time between image presentation and voice onset when the participant begins naming the image.

Photographs are often considered more ecologically valid than line drawings because they include rich detail, such as color and surface texture. While photographs represent a specific instance of an object, line drawings, which lack surface detail, tend to depict a generic class or prototype of the concept [4]. However, the richness of photographs can increase naming variability; while photographs facilitate recognition, they also elicit more alternative names than line drawings [3,5–7]. It is well established that images with high name agreement are named more quickly than those with low name agreement [8–14]. In their Bayesian meta-analysis of 18 line-drawing norming studies, Perret & Bonin [15] showed that image and name agreement, imageability, age of acquisition (AoA), and conceptual familiarity reliably predicted naming latencies, whereas visual complexity and word length did not. They also noted the inconsistent inclusion of latency measures across norming datasets, which constrains cross-study comparisons.

Recent work emphasizes the value of including RT norms alongside naming agreement measures. For example, one study [16] showed that recognition RT, reflects not only modal name but also the distribution of plausible alternatives. Other studies demonstrate that images with more surface detail are associated with faster recognition [17,18]. Given that photographic images tend to elicit a wider range of alternative names, RT norms would be a valuable addition to image norming studies. However, unlike word frequency, word length or AoA, RT norms are particularly sensitive to the age of the normative sample. Thus, naming RTs can reflect age-related changes in lexical retrieval, offering insight beyond binary correct/incorrect responses.

Despite these insights, most normative studies on images of common objects rely on young, highly educated participants [2]. This limits their relevance for aging populations, where lexical retrieval may slow and at times decline in accuracy [19–26]. Given that image naming is a standard assessment for word-finding deficits in conditions such as dementia [27–31], normative data sets specific to middle and older adults are essential for distinguishing typical aging from pathological decline.

The Bank of Standardized Stimuli [1,3] is one of the most widely used photographic stimulus banks, containing 1,410 normed photographs of everyday objects. It includes norms for name agreement, familiarity, category, visual complexity, object agreement, viewpoint agreement, and manipulability [1,3]. However, these norms are based primarily on responses from younger adults while norms for adults age 40 + are largely absent. In addition, name agreement and modal name in the BOSS studies are based on written naming tasks, and naming RTs were not included. The purpose of the current study is to provide normative data for a subset of BOSS images based on a sample of adults age 40 and older. Our dataset includes measures of name agreement, modal names, alternative names, the H-statistic, and naming RTs. By offering both RT data and the range of alternative names produced, this dataset addresses a critical gap in image norming and supports both experimental research and clinical applications. The complete dataset is available in the supplemental materials.

## Methods

Participants: Eighty-two participants were recruited. Initial local recruitment via snowball sampling was switched to a recruitment method that involves online recruitment via ResearchMatch [32] to ensure a more geographically and demographically diverse normative sample. All participants were provided with an informed study participation letter approved by the Medical College of Wisconsin Institutional Review Board. The data was analyzed anonymously. No personal identifying information was collected or saved, and audio recordings were deleted after responses were transcribed and scored. All individuals were native speakers of English and self-reportedly free of neurological diagnoses (i.e., no reported diagnoses of mild cognitive impairment, dementia, etc.). Access to the internet and a personal computer in a quiet space of their home were required for participation in this study, in addition to being at least 40 years of age. The mean age of our full cohort was 57.4 years (SD = 11.3; range = 40–83 years) and mean education was 16.3 years (SD = 2.3, range = 11–20 years). Five participants were ambidextrous, 7 were left-handed and the remaining were all right-handed. Sixty-one participants (74.4%) self-identified as White, 11 (13.4%) as Black or African American, 3 (3.6%) as Asian and 7 (8.5%) as belonging to more than one race. Six (7%) participants self-identified their ethnicity as Hispanic or Latino. See Table 2 for a descriptive summary.

**Table 2:** Demographics of the Full Cohort and Separated by Age Groups.

		Age Groups		
	All	40–50	51–65	66+
N	82	24	34	24
Age	57.4 (11.33)	43.6 (3.15)	58.3 (4.08)	69.9 (7.40)
Education	16.3 (2.33)	16.6 (2.16)	16.1 (2.31)	16.2 (2.57)
Sex	55 females 27 males	18 females 6 males	23 females 11 males	14 females 10 males
Handedness	5 ambidex 7 left 70 right	1 ambidex 3 left 20 right	4 ambidex 1 left 29 right	0 ambidex 3 left 21 right
Race	61 White 11 Black 3 Asian 7 Multiracial	16 White 4 Black 1 Asian 3 Multiracial	24 White 4 Black 2 Asian 4 Multiracial	21 White 3 Black 0 Asian 0 Multiracial
Hispanic or Latino Ethnicity	6	4	1	1

Standard deviation in parenthesis; ambidex = ambidextrous.

Stimuli: A total of 600 photographs were selected from the BOSS, representing a variety of object categories. The majority of the photographs were of nonliving objects; see S1 Table for distribution of images across semantic categories. Stimuli were chosen to create a varied and representative set of stimuli, capturing a range of object types and perceptual features such as familiarity, visual complexity, and name agreement. Selection was further guided by practical considerations, including session length and minimizing participant fatigue, to support both experimental and clinical applications. The stimuli were originally selected for a separate speech therapy intervention. To avoid overlap with items used in the pre- and post-assessment (Snodgrass and Vanderwart [33] set used in the Language Imaging Lab Aphasia Battery Picture Naming assessment and word reading assessment [34], any BOSS images corresponding to assessment items were excluded to prevent interference. This constraint affected category distribution, as some categories were overrepresented in the assessment set and thus had fewer items in the final stimulus selection. This approach produced a dataset independent of our clinical naming assessment tool, providing normative data for a large corpus of items representative of older adults. Each image consisted of a color photograph presented on a white background with no contextual cues, consistent with the BOSS’s standardized formatting. All images were standardized to a resolution of 2000 × 2000 pixels. Following an initial review of data from approximately 25% (n = 22) of participants, 10 images were replaced due to consistently low naming accuracy and frequent misidentification (e.g., a photo of sandpaper was commonly mistaken as cheese; a balcony was perceived as a piece of cake, a cage, or a prison). These problematic items were substituted to ensure clearer object recognition across the sample. For completeness, all items – including the 10 replaced images – are reported in the supplemental file. However, note the lower number of participants that these normative values are based on for those select images. They were excluded from any subsequent data analysis in this report.

For detailed stimulus creation procedures, see the original study [3]. A list of stimuli used in the current study with traditional BOSS norms for name agreement and H value is provided in the supplemental files.

Procedure: Data collection was conducted synchronously and virtually through a secure video-conference platform (Zoom), typically in participants’ homes. This study was initiated during the COVID-19 pandemic lockdown, which necessitated minimizing in-person contact and limited the feasibility of in-lab data collection. The experimenter controlled the stimulus presentation and took notes regarding unexpected events during data collection (e.g., occasional audio system failures or interruptions). Participants were instructed to name the pictures as quickly and accurately as possible. Each trial began with a blank screen displaying a central crosshair for 400 milliseconds (ms), followed by a beep tone presented simultaneously with the onset of a single image. After the participant responded, the experimenter pressed the space bar to proceed to the next trial, beginning with the crosshair. The maximum duration for response was 4,000 ms after which the next trial would begin. Scheduled breaks were provided after every 100 trials. Audio recordings were made on both the participant and experimenter ends and the higher-quality recording was used for offline data scoring of RT and naming accuracy. Analysis: A custom Python script was developed to facilitate standardized scoring of naming responses. The script allowed evaluators to code responses using a binary system: a “1” indicated a correct response matching the modal name from the BOSS norms, while a “0” denoted a response that did not match the modal name. If a response did not match the modal name, the participant’s response was manually entered and evaluated. Correct alternative responses; those differing from the modal name but still considered accurate (i.e., fishing pole, fishing rod), were also marked as “1” during preliminary analysis. If an incorrect response was recorded, an additional prompt would ask for the type of error made. Semantic errors were indicated with a “S,” phonological errors with a “F” or “FN” (formal word or formal nonword), mixed (semantic and phonological) errors with ‘M’, no response trials with an “O” for omission, and other unrelated errors with U or UN (unrelated word or nonword). All evaluators were native speakers of English who completed training prior to conducting this task. Discrepancies between evaluators were mediated by a third researcher and a consensus was reached. Response Categorization: Individual responses were collated into master files for every image. Evaluators then inspected, verified and, where necessary, corrected the initial correct/incorrect classifications. In addition, a categorical coding system was devised to categorize responses into acceptable and unacceptable responses, based on procedure described previously [6]. Acceptable responses were categorized into correct (modal name), synonymous (semantically equivalent to the modal name), sub-ordinate or supra-ordinate responses.

Three examples illustrate this approach. For an image with the modal name *video tape*, the response *VHS tape* would be categorized as a synonymous and thus acceptable response. For the modal name *eggplant*, a supraordinate response of *vegetable* would be marked as correct. Likewise, for *skate* as the modal name, *ice skate* would be accepted as a subordinate response. Unacceptable responses included idiosyncratic responses, actions, misconceptions, and names that conveyed incorrect information. An example is a response of *hockey table* for the presented image of *foosball table*. Two evaluators received extensive training on the coding procedure and completed the analysis after they reached a 100% inter-rater agreement on practice trials. Ongoing inter-rater reliability checks were conducted across 20% of the data (120 trials), revealing consistently high inter-rater agreement of 90%.

RT measurement: The RTs were also recorded and scored off-line using a custom-built Python script. The script estimated RT by calculating the latency between stimulus onset and the onset of the participant’s spoken response. For each trial, speech envelopes were derived from the corresponding auditory waveform, containing two waveforms: the first marked the onset beep, synchronized with the presentation of the picture, and the second marker corresponded to the onset of the participant’s spoken response. The onset markers were manually adjusted as needed. Due to the presence of occasional background noise, the determination of the response onset required manual inspection. Trained raters identified the first clear speech signal following the beep and used this as the response onset marker. This manual process ensured more accurate RT estimates than automated detection alone could provide.

The speech envelope was derived from the trial-wise recordings by taking the magnitude (absolute) of the Hilbert Transform and filtering it to obtain low frequency components (4–20 Hz low-pass). Samples of the trial speech envelope are shown in S1 Fig.

H value was computed using the following formula from [10]:


$$\begin{array}{c} \text{H }=\sum_{i=\text{1}}^{k}P_{i}{\text{log}}_{\text{2}}\left(\frac{\text{1}}{P_{i}}\right) \end{array}$$

Where k = number of alternative acceptable names for each image, P_i_ = proportion of participants producing each of the acceptable name. Thus, for an image with a unique name with no other acceptable alternative name the H value is 0. H value increases as a function of the number of alternative responses [10].

Modal Name and Name Agreement: For each image, responses were analyzed after excluding “don’t know” (DK) and tip-of-the-tongue (TOT) responses. DK responses included both omissions and explicit verbal reports of “I don’t know.” Due to the oral response format, it was not possible to reliably distinguish between participants who did not know what the object was or recognized the object but did not know the name and tip of the tongue responses. Some hesitations or indications of difficulty in retrieval could reflect a TOT state, but they could also indicate that the participant simply did not know the name. Therefore, these responses were conservatively treated as a single DK/TOT category and excluded further. The name given by the highest percentage of participants is the modal name. The percentage of participants who provided the modal name is the name agreement.

Alternative names were assessed for spelling errors and singular/plural responses were collapsed into a single alternative/acceptable response before computing H value and naming agreement metrics. For example, coders may have used different spellings for the same verbal response (e.g., mit and mitt), which were both considered acceptable but as a single alternative name. Example for a plural is ice-skate and ice-skates, which were both considered acceptable but as a single alternative name. These corrections ensured that H values were not inflated due to duplicate responses.

RT data were cleaned by removing trials with slow (RT > 4,000 ms) responses, which fell outside the response window. RT data for trials with omissions, i.e., those with no responses, were scored as 0, which were also removed from further RT analyses. After this first round of data cleaning, RTs within 3 SDs of the participant mean were retained for the final analysis. For analysis of the accuracy data, all trials were included, i.e., all with slow responses as well as omissions. For speed-accuracy tradeoff analysis, omission trials with RT of 0 ms were excluded.

All statistical analyses were conducted in R and RStudio [35] using nonparametric permutation-based tests because assumptions for parametric tests, particularly that of normality, were not met. Computational norms *across participants* (collapsed across modal names; participant-level analysis) were analyzed using permutation-based ANCOVA (5000 randomizations) using the *permuco* R package [36] to examine differences across age groups after controlling for education levels. The participant-level analyses focused on group differences in the RT and accuracy data, to evaluate age-related changes in these response metrics. Norms *across modal names* (collapsed across participants; item-level analyses) were analyzed using permutation-based ANOVA (5000 randomizations) constrained within modal names (shuffling age groups) to assess item-level effect of age groups using custom code and the *coin* R package [37]. These analyses focused on H value and name agreement metrics, which are derived for each item by summarizing response characteristics across participants (e.g., proportion of participants producing each of the acceptable name for a given item). RT and accuracy data are also compared using item-level analyses for completeness. To control for multiple comparisons, p-values were adjusted using the Holm’s method. Adjustments were applied across 3 performance measures (accuracy, RT, correct RT) at both the participant and item levels, and across 2 item-level measures (H value and name agreement). The effect sizes are reported for significant results using partial eta-squared (ƞ^2^) with 95% confidence intervals (CI) derived using bootstrapping. Post-hoc pairwise comparisons were also conducted using permutation testing for participant-level (repeated measures) and item-level (repeated measures blocked by modal name) effects between age groups, both corrected for multiple comparisons using the Holm’s method. Finally, internal consistency was computed for name agreement and H values via iterative split-half analysis (1000 iterations). Eighty-two participants were randomly divided into 2 equal sized groups (41 in each group) and then correlations were computed between the sampled groups using Spearman’s rho.

## Results

### Participants

Eighty-two (55 female; 27 male) participants completed the study resulting in 47925 responses for 600 images. Due to technical difficulties during data collection and error in RT measurement script, 1275 responses were missing (2.6% missing data); the distribution of missing data across items and participants is provided in S2 Fig. Participants were divided into 3 groups according to the following age ranges: 40–50, 51–65, 66 + . Descriptive statistics for demographical information by age groups are provided in Table 2. Mean education (p = .20) was not different across age groups.

**Fig 1:**
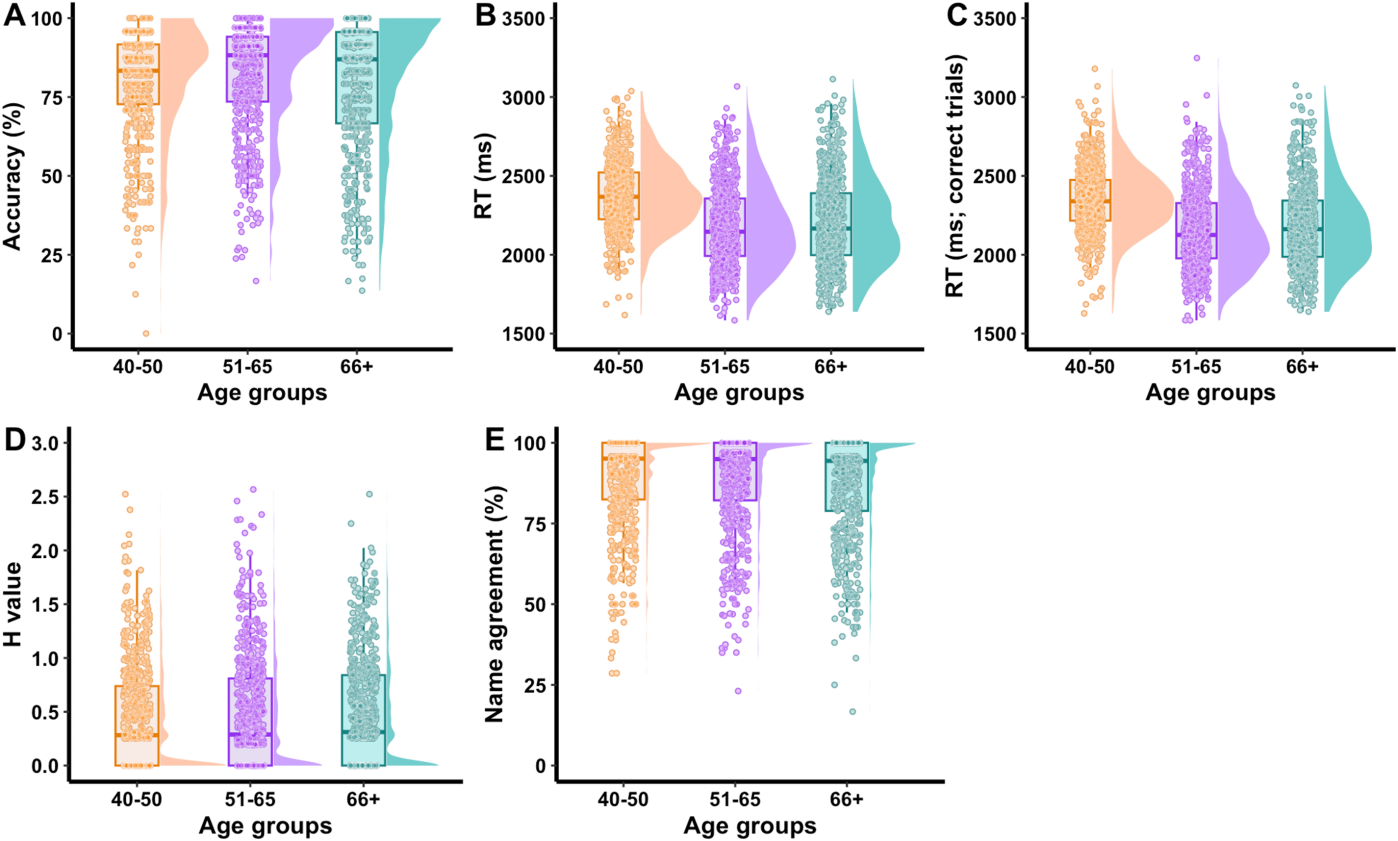
Participant-level accuracy and RT. Participant-level accuracy (A) and RT (B) data collapsed across modal names. No differences in accuracy or RT were significant across age groups, except for RTs derived from correct trials (C). Age group 40–50 was the slowest to respond.

Mean accuracy for the full cohort was 80.5% (SD = 16.4%) and the mean RT was 2264 ms (SD = 253). Semantic and omission errors were the most common error types, respectively comprising 54.5% and 45.0% relative to all errors produced in the full cohort; for error type distribution across age groups see S2 Table. Phonological and unrelated errors comprised a small proportion (.1% and.4%). The accuracy was numerically the highest (81.9%, SD = 16.8) and RTs were the fastest (2202 ms, SD = 283) in the 51–65 group. However, the main effect of age group was not significant for accuracy, F(2, 78) = 1.0, permutation p = .383, or for RT from all included trials, F(2,78) = 2.72, permutation p = .070. The main effect of age group on RT from correct trials reached nominal significance, F(2,78) = 3.28, p = .045, partial η² = .08, 95% CI [.01,.25]; however, this effect did not survive correction for multiple comparisons (corrected p = .135). Post-hoc pairwise tests indicated that the 40–50 group tended to respond more slowly (M = 2358 ms, SE = 45) than the 51–65 group (M = 2167 ms, SE = 40; p = .081). No other group differences reached significance (ps > .10). See Table 3 for descriptive summaries of naming performance and Fig 1 for participant-level accuracy and RT plots.

**Table 3:** Descriptive Summary of Computational Norms for Image Naming.

		Age Groups		
	All	40–50	51–65	66+
*Accuracy (%)	80.5 (16.4)	79.6 (16.7)	81.9 (16.8)	79.1 (19.6)
*RT (ms)	2264 (253)	2392 (232)	2202 (283)	2228 (295)
*RT (correct trials only; ms)	2229 (131)	2358 (221)	2167 (265)	2192 (273)
H value	.538 (.552) Mdn = .352	.430 (.502) Mdn = .282	.476 (.541) Mdn = .288	.478 (.523) Mdn = .311
Modal name agreement (%)	87.4 (15.2) Mdn = 94.6	88.7 (15.2) Mdn = 95.1	88.2 (15.4) Mdn = 95.0	87.2 (16.1) Mdn = 94.4

*these were computed by first collapsing across participants within each age group and modal name, and then averaging across the modal names; Standard deviation in parenthesis represents variability across modal names (and not participants); Mdn = median.

**Fig 2:**
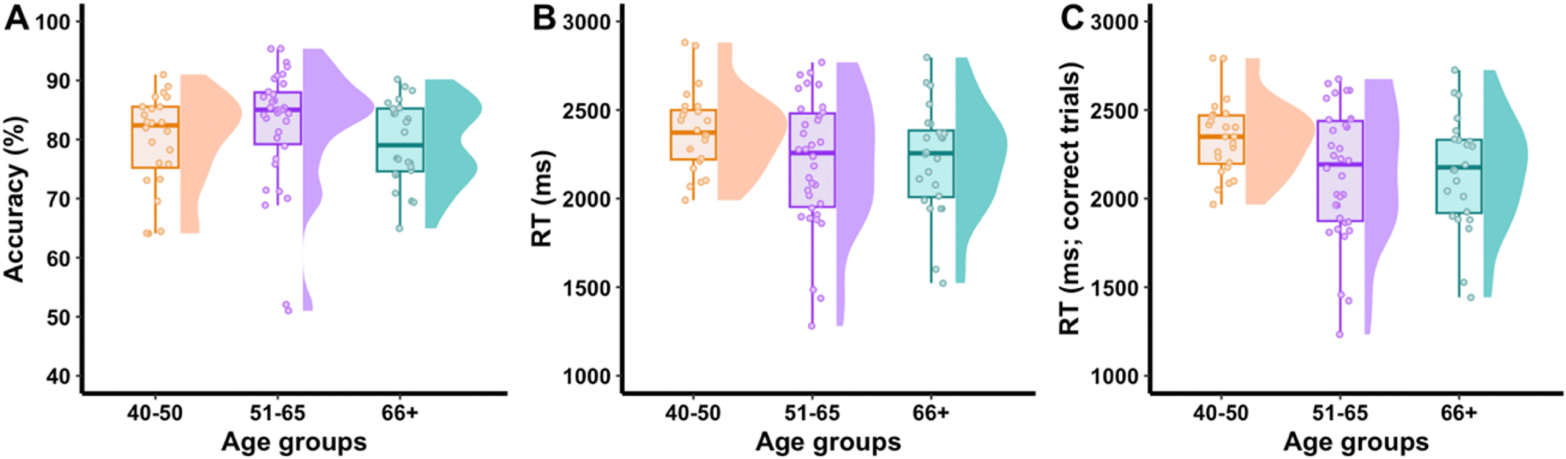
Item-level accuracy, RT, H-value, name agreement. Item-level accuracy (A), RT (B, C), H value (D) and name agreement (E) data collapsed across participants and blocked by modal names for analyses. Age group 40–50 was the least accurate and slowest to respond. Yet that group produced responses with the fewest alternatives and highest name agreement, particularly compared to the 66 + group.

For item-level accuracy (Fig 2), the main effect of age group was significant, F(2,1170) = 19.3, permutation p < .001, corrected p < .001; partial ƞ^2^ = .033, 95% CI [.020,.055]). Post-hoc permutation test indicated that the 51–65 group outperformed both other groups (both p < .001) with no significant difference between the 40–50 and 66 + groups (p = .350). For RT data, the main effect of age groups was significant, F(2,1170) = 376.5, permutation p < .001, corrected p < .001; partial ƞ^2^ = .64, 95% CI [.53,.80]. The post-hoc comparisons indicated that the 40–50 group responded more slowly compared to both other groups and the 66 + was slower than the 51–65; similar patterns were found for RT from correct trials, F(2, 1169) = 337.5, permutation p < .001, corrected p < .001; partial ƞ^2^ = .58, 95% CI [.46,.73].

Only item-level analyses were conducted for norms related to characterizing the modal names, i.e., the H value and name agreement. The mean H value was 0.459 (SD = 0.521) for the full cohort. The mean rho from the iterative split-half analysis after 1000 iterations indicated an internal consistency of 0.83 for H value and 0.84 for name agreement, both in the acceptable range. Permutation-based ANOVA with blocked modal names indicated a significant main effect of age group, F(2, 1162) = 7.17, permutation p < .001, corrected p < 0.001, partial ƞ^2^ = .012, 95% CI [.003,.030]. To verify that these results were not biased by differences in sample sizes across age groups, we re-analyzed these data with equal sample sizes via subsampling of the 51–65 group. These additional analyses indicated a minimal impact of sample size differences on H value in our cohort (see Supplementary Additional Analysis). Post-hoc permutation tests indicated that the H value was lower in the 40–50 group compared to both 51–65 (p = .004) and 66+ (p = .004) groups and that the 2 older groups were not different from one another. The youngest group produced unique names with fewer alternatives compared to both 51–65 and 66 + groups. For name agreement, a significant main effect of age group, F(2, 1162) = 5.8, permutation p = .005, corrected p = .005; partial ƞ^2^ = .010, 95% CI [.001,.027]) was found, and post-hoc tests indicated that name agreement was higher in the 40–50 group than the 66 + group (p = .007). The youngest group had the highest name agreement compared to the oldest group. No other group differences were significant. Overall, item-level analyses indicate that the youngest age group produces responses that are relatively less accurate and slower compared to the older groups, yet with fewer alternative responses (H value) and higher name agreement. Although the effects are significant, the magnitude of differences, except for RT differences, across age groups is small.

**Fig 3:**
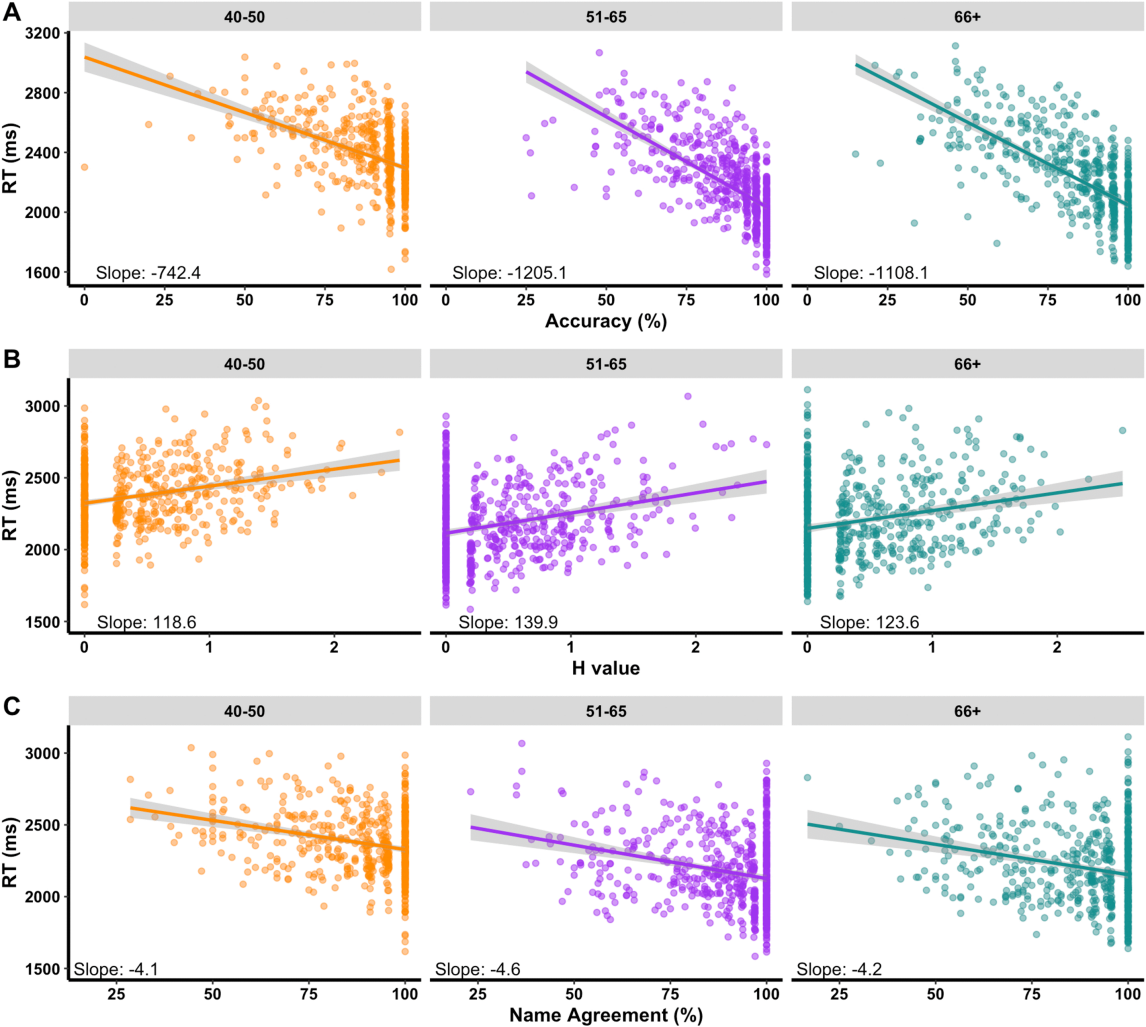
Relationships between RT and other norms. (A) speed-accuracy tradeoff is observed in all age groups with slightly weaker relationship (shallower slope) for the 40–50 age group. (B, C) RTs are longer with higher H values and lower name agreement.

The relationships between norms in each age group were also explored: RT vs. Accuracy, H value and name agreement (Fig 3). As expected, significant speed-accuracy tradeoffs were found in all age groups (Fig 3A) with slightly weaker relationship for 40–50 group (slope = −742) compared to the other groups (slopes > 1100). As expected, RTs increased with higher H values and lower name agreement (Fig 3B, C), across all age groups.

**Fig 4:**
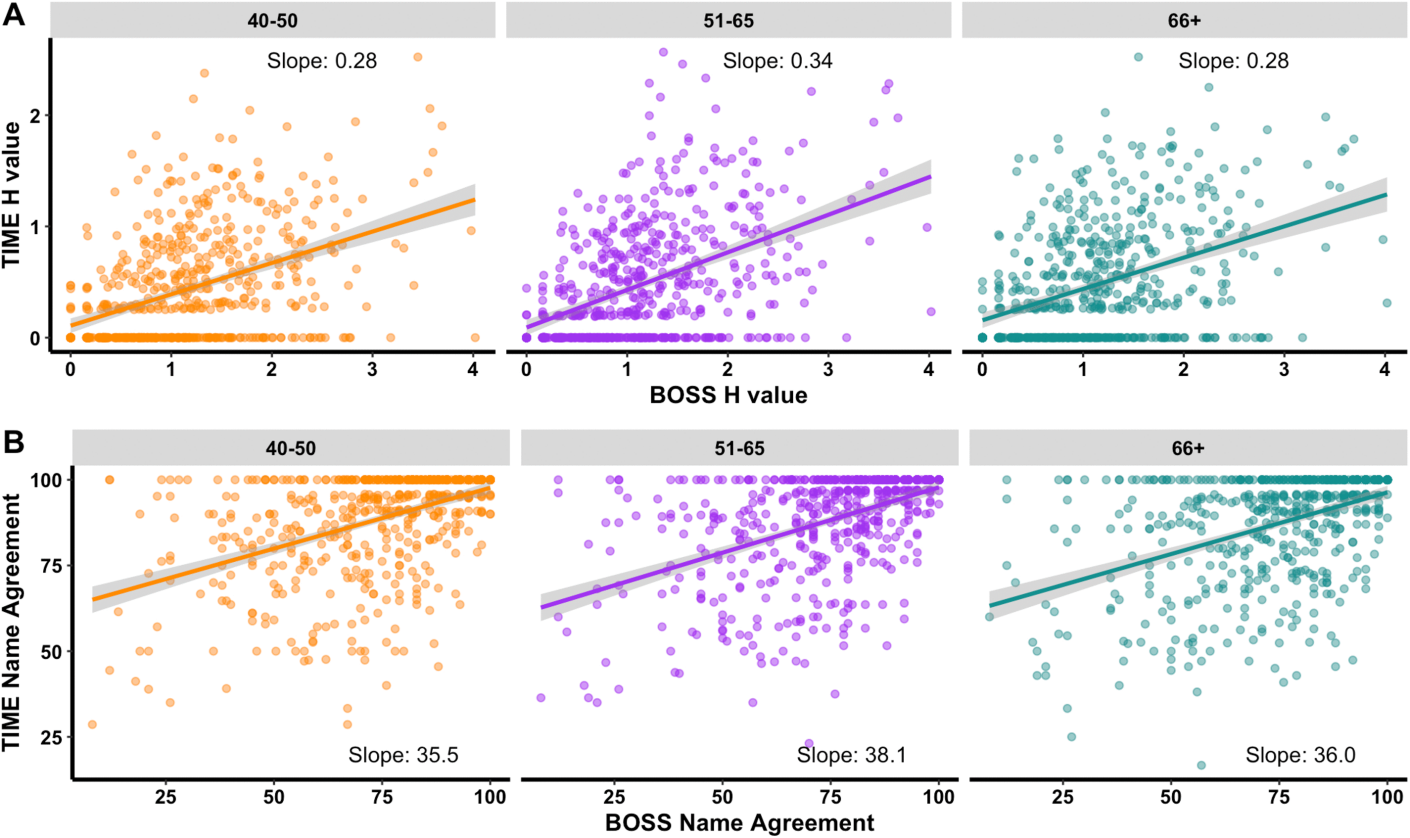
Correlations between BOSS and TIME. Significant correlations between BOSS and TIME H value (A) and name agreement (B) norms, in all age groups.

Additionally, H value (rho = .45,.49,.41, respectively for 40–50, 51–65, and 66 + groups) and name agreement (rho = .49,.51, 44) between BOSS norms and those from our cohort (separated by age groups) were highly correlated with significant positive relationships (p < .001) Fig 4.

## Discussion

Lexical retrieval is a core aspect of language processing that shows remarkable resilience across the adult lifespan. In this study, we provide normative data on image naming performance for adults aged 40–83 years, extending prior work based largely on younger samples. Across cohorts, naming accuracy and name agreement were high, and H values low, suggesting stable lexical access and semantic organization. Response times showed an unexpected nominal pattern, with younger adults tending to name pictures more slowly than older participants; however, this effect did not remain significant after correction for multiple comparisons. This reversal of the expected age trend may reflect sampling differences or contextual factors inherent to web-based data collection. Together, these results underscore both the robustness of lexical retrieval in healthy aging and the methodological sensitivity of RT measures.

Across the full cohort, mean naming accuracy was 80.5% (SD = 16.4%) and mean response time (RT) was 2264 ms (SD = 253). Accuracy was highest and responses fastest in the 51–65 age group, though age effects on accuracy were not statistically significant. Nominal age-related differences were observed in RTs, with the youngest group (40–50 years) showing slower RTs than both older groups; however, this effect did not remain significant after correction form multiple comparisons. Item-level analyses revealed small but consistent age effects in name agreement and H value: younger adults showed greater naming consistency (higher name agreement, lower H values), whereas older participants produced slightly more variable responses. Although statistically significant, these differences were small in magnitude, suggesting that lexical retrieval remains stable through midlife and older adulthood.

These findings align with previous research showing that while aging is associated with slower processing speed and occasional word-finding difficulties, the integrity of lexical-semantic representations is generally maintained [26,38]. Although the overall pattern suggests preserved language function in healthy aging, the nominal slowing observed among younger adults departs from prior literature showing age-related increases in naming latency. Contrary to previous reports of age-related slowing [19,20,24,25], the youngest cohort in our sample exhibited longer RT than older adults; however, this pattern should be interpreted cautiously given that the corrected participant-level RT analysis was not statistically significant. This discrepancy likely reflects methodological or sampling factors rather than cognitive decline. Younger participants may have been less engaged during remote testing, whereas older volunteers, particularly those recruited through ResearchMatch, may represent a more motivated, high-functioning subgroup. A comparison of naming response data from the present study with previously published datasets is provided in Table 4.

**Table 4:** Summary of present study norms and other recent image norms studies.

Study	Participant Age/N	Stimuli N	Modal Name Agreement	H Value	Language	RT	Accuracy
Present Study	57.4/82	600	87.4 (15.2)	.54 (.55)	English (US)	2264	80.5 (16.4)
Van Hoef et al. [16]	37.2/60	200	66.25 (22.02)	1.38 (.82)	English (UK)	1055	n/a
Brodeur et al., [1,3]	29.4/81	1469	59.00 (25.00)	1.86 (1.08)	English (CAN)	n/a	n/a
Krautz & Keuklers [39]	22.2/40	1547	79.00 (23.00)	.69 (.70)	German	1252	80 (22.0)

n/a = not available.

Only subtle changes were observed in the cohort of 66 + . The mean H value was almost identical to the cohort of 51–65 (.472 vs.476) suggesting stabile and consistently low variability in name agreement with increasing age. While expressive language skills have been shown to decline with age, often characterized by simpler language and more frequent instances of tip-of-the-tongue moments [38,40], only a subtle, nonsignificant decrease in naming accuracy compared to the two younger cohorts was observed in the current study.

A correlation analysis of the collected norms revealed the anticipated trade-off between RT and accuracy across the three cohorts. Greater name agreement and lower H values were also associated with faster RTs. Notably, RT variability persisted even for items with 100% name agreement and H values of 0. The sources of this variability can be explored further in the shared data set, which allows item selection based on RT and other psycholinguistic variables. This study used a subset of BOSS photographs. While we provide the original BOSS norms in the supplemental file, comparisons between the norms of the current study and previously published BOSS norms [1,3] should be considered cautiously due to difference in sample characteristics (e.g., age, geographic region and cultural background) and methodological differences in eliciting naming responses. The original BOSS studies elicited written naming responses in a controlled laboratory environment while the current study relied on oral naming responses elicited in a virtual study with participants in their home environments. In addition, we did not collect perceptual ratings of visual complexity, object agreement or viewpoint agreement or familiarity. Neither did we elicit category agreement. However, the oral naming allowed us to collect RT data, a novel contribution to the BOSS norms, in addition to expanding the age range of the BOSS normative sample.

Future analyses could examine the qualitative aspects of alternative names and erroneous responses to provide more fine-grained analysis of image naming performance in older adults. For example, sources of error productions could be based on visual misconceptions (coconut –kiwi) or lexical-semantic retrieval deficits (e.g., naming actions that describe the depicted concept as a form of circumlocution). Understanding emerging patterns of lexical retrieval deficits will facilitate better definitions of healthy and impaired lexical retrieval performance in older adults. Further, developing parameters for stimulus selection guidelines incorporating variables that capture naming uncertainty through integrating the H statistic, RT and psycholinguistic variables will allow for better stimulus control while preserving a range of naming difficulty levels.

A limitation of this study is the absence of a cognitive screener, as undetected cognitive impairments could have influenced the results, particularly given that the likelihood of cognitive impairment increases with age. Consequently, the normative data should be interpreted with caution. Although the sample showed relative stability across age groups and small effect sizes for differences in naming accuracy and H values, these patterns cannot confirm that participants were free of cognitive impairment. Future studies incorporating standardized cognitive assessments are needed to validate these norms and ensure that they reflect cognitively healthy populations.

Given the limited demographic information available, additional confounding variables may have contributed to the observed results. Although we aimed to recruit a diverse and representative sample, participants were likely a relatively high-functioning, well-educated, and technologically proficient group with adequate internet access. Recruitment through a program called ResearchMatch, which connects research-interested volunteers with potential studies, may have introduced motivation-related biases. Collectively, these factors could have contributed to the unexpected RT differences.

At the same time, the selected recruitment mechanism and virtual data collection provided notable advantages, enabling inclusion of participants across a wider geographic area and reducing common barriers such as travel and scheduling constraints that often limit in-person studies. The online format may also have social desirability effects, as the participants completed tasks in familiar surroundings rather than formal testing settings, potentially yielding more ecologically valid data [41]. Nevertheless, differences in recruitment and data collection compared to previous laboratory-based studies of aging and picture naming may partially account for discrepancies in findings. Lastly, although this study provides normative data for adults aged 40 and above, data were collected exclusively in the US and in English, limiting cross-cultural generalization.

While methodological and sampling limitations should be acknowledged, this study expands the scope of existing image naming research by providing normative data for adults aged 40 years and older. In doing so, it addresses a longstanding gap in psycholinguistic and clinical literature, where most norming data have been derived from younger adult samples. Prior BOSS norms [1,3] and most imaging naming studies have focused primarily on younger adults aged 18–35 (see Table 4 and Souza et al. [2]. The inclusion of middle-aged and older adults enables a more comprehensive understanding of lexical retrieval across the adult lifespan.

The cohort groups were motivated by clinical considerations. Age 65 is commonly used as the cutoff for distinguishing early-onset from late-onset dementia, making it a meaningful threshold for characterizing normative cognitive-linguistic function. Most neurodegenerative conditions, including Alzheimer’s and Parkinson’s disease, occur more frequently after age 65, while others, such as primary progressive aphasia, Huntington’s disease and amyotrophic lateral sclerosis often appear earlier in life. Consequently, normative data on unimpaired cognitive functioning in adults in their 40s, 50s, and early 60s are essential for establishing benchmarks of typical performance. Furthermore, recent epidemiological evidence highlights a rise in stroke prevalence among adults aged 18–64 years, across multiple sociodemographic groups, including differences by sex, race and education level [42]. Consequently, normative data for cognitive-linguistic functioning in these age ranges is of clinical relevance. While this study applied a clinically motivated age grouping, alternative approaches to data stratification could be explored in future analyses.

## Conclusion

In conclusion, norms for adults aged 40 + are imperative for advancing psycholinguistic research on lexical retrieval and RT. The present findings contribute novel insights into both the stability and variability of imaging naming performance in older adults. Findings support both theoretical models of aging and practical applications for enhancing communication and cognitive health in aging populations. The corpus of images is large, including 600 photographs, ranging widely in corresponding psycholinguistic variables. By including measures of RT and alternative names produced, this dataset enables researchers to more comprehensively evaluate and select photographic stimuli for use in experimental and clinical settings.

The norms from the current study can be accessed via the Open Science Framework (OSF) at https://osf.io/mhpbn/?view_only=c21c43adcfce4490be514375013d9575.

Two sets of aggregated data, along with cohort demographics, are available in the OSF repository. The first set (Supp1) includes modal names, H values and name agreement norms from BOSS as well as new norms computed in the current study aggregated by *all* participants, or separated by the age groups, including accuracy, RT and RT from correct trials. Together these norms can be used to compute a composite score to derive an index of retrieval difficulty and for grouping modal names into easy, intermediate and hard categories. The second set (Supp2) of data includes summaries of item-wise number and responses associated with correct and incorrect trials. The files also include codes for the error types [43]. Finally, demographics for all participants are included in Supp3_Demographics file.

## Supporting information

S1 Fig Speech envelope example. Samples of speech envelopes for two trials corresponding to correct (Example 1) and incorrect (Example 2) responses.
(DOCX)

S2 Fig Distribution of missing data. The number of missing items (images) across participants (A) and the number of participants with missing data across items (B).
(DOCX)

S1 Table The number of images (N) per semantic category. Sixteen semantic categories were included from the BOSS database.
(DOCX)

S2 Table Error type proportion. Proportion (%) of each error type relative to all errors produced within each age group.
(DOCX)

S1 Additional Analysis Item-level H value analysis. (DOCX)
