# Supplementary material for: Timed image naming evaluation for adults (TIME) using BOSS images
Source: PLoS One. 2026 Mar 9;21(3):e0341774. doi: 10.1371/journal.pone.0341774 (PMC12970895; doi:10.1371/journal.pone.0341774)
Supplement: S1 Fig — Samples of speech envelopes for two trials corresponding to correct (Example 1) and incorrect (Example 2) responses. (DOCX) [file pone.0341774.s001.docx]

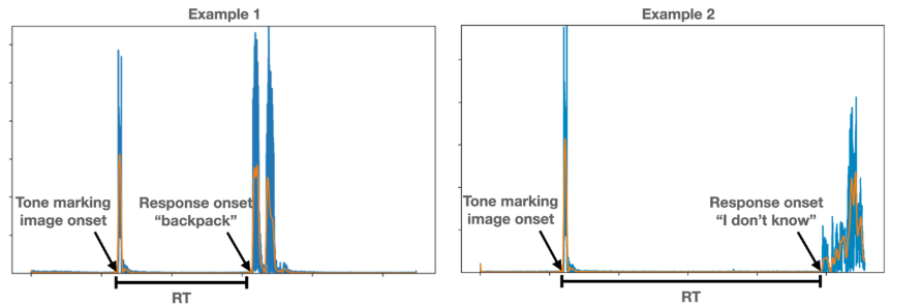

**Supplementary Figure 1.** Samples of speech envelopes for two trials corresponding to correct (Example 1) and incorrect (Example 2) responses.
