# Supplementary material for: Timed image naming evaluation for adults (TIME) using BOSS images
Source: PLoS One. 2026 Mar 9;21(3):e0341774. doi: 10.1371/journal.pone.0341774 (PMC12970895; doi:10.1371/journal.pone.0341774)
Supplement: S2 Fig — The number of missing items (images) across participants (A) and the number of participants with missing data across items (B). (DOCX) [file pone.0341774.s002.docx]

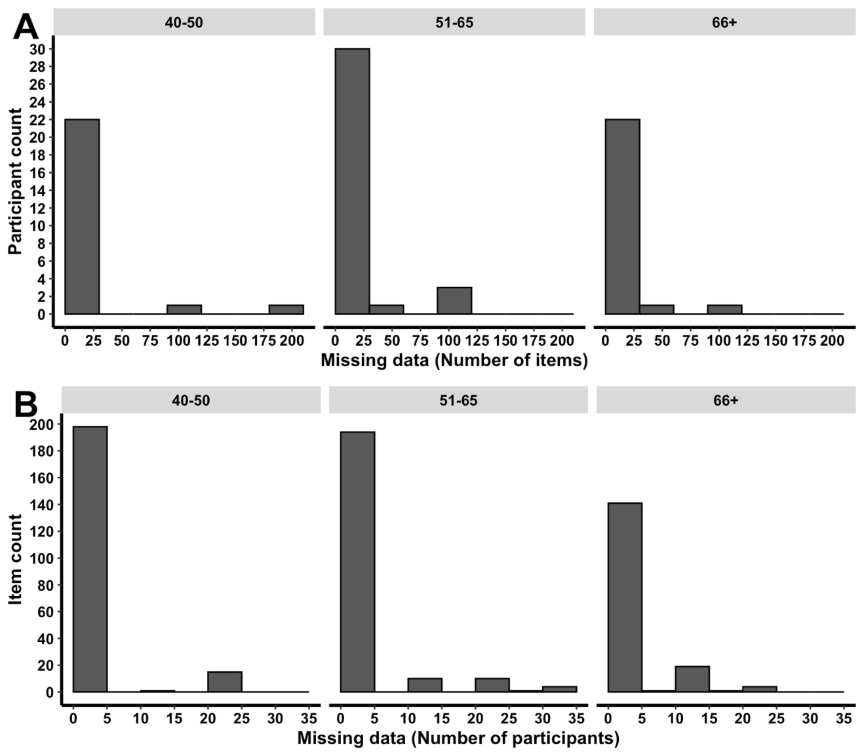


**Supplementary Figure 2.** Distribution of missing data. The number of missing items (images) across participants (A) and the number of participants with missing data across items (B). Distribution across groups and items appear to be uniform, with minor differences for 51-65 group for the number of missing items, likely due to a slightly larger N (of 34) compared to the other groups (N of 24 each).
