# Supplementary material for: Timed image naming evaluation for adults (TIME) using BOSS images
Source: PLoS One. 2026 Mar 9;21(3):e0341774. doi: 10.1371/journal.pone.0341774 (PMC12970895; doi:10.1371/journal.pone.0341774)
Supplement: S1 Table — Sixteen semantic categories were included from the BOSS database. (DOCX) [file pone.0341774.s003.docx]

Sixteen semantic categories were included from the BOSS database. The number of images per category is provided in Supplementary Table 1.

| **Supplementary Table 1.** The number of images (N) per semantic category | | |
| --- | --- | --- |
| **Category 2** | **Category 1** | **N** |
| Animal | Living | 73 |
| Body part | Body part | 5 |
| Building | Nonliving | 46 |
| Electronic | Nonliving | 23 |
| Fashion | Nonliving | 38 |
| Food | Living | 70 |
| Furniture | Nonliving | 16 |
| Household item | Nonliving | 31 |
| Instrument | Nonliving | 14 |
| Natural element | Natural element | 7 |
| Plant | Living | 11 |
| Sport | Nonliving | 40 |
| Tool | Nonliving | 170 |
| Toy | Nonliving | 20 |
| Vehicle | Nonliving | 24 |
| War item | Nonliving | 12 |
