# Supplementary material for: Timed image naming evaluation for adults (TIME) using BOSS images
Source: PLoS One. 2026 Mar 9;21(3):e0341774. doi: 10.1371/journal.pone.0341774 (PMC12970895; doi:10.1371/journal.pone.0341774)
Supplement: S2 Table — Proportion (%) of each error type relative to all errors produced within each age group. (DOCX) [file pone.0341774.s004.docx]

| **Supplementary Table 2. Proportion (%) of each error type relative to all errors produced within each age group.** | | | | |
| --- | --- | --- | --- | --- |
|  |  | **Age groups** | | |
| **Error Types** | **Error Code** | **40-50** | **51-65** | **66+** |
| Formal Word | F |  | 0.03 | 0.07 |
| Formal Nonword | FN | 0.07 | 0.06 | 0.07 |
| Mixed | M |  | 0.09 |  |
| Omission | O | 54.64 | 41.57 | 39.63 |
| Semantic | S | 45.22 | 57.63 | 59.80 |
| Unrelated Word | U | 0.07 | 0.43 | 0.28 |
| Unrelated Nonword | UN |  | 0.20 | 0.14 |
