# Supplementary material for: Timed image naming evaluation for adults (TIME) using BOSS images
Source: PLoS One. 2026 Mar 9;21(3):e0341774. doi: 10.1371/journal.pone.0341774 (PMC12970895; doi:10.1371/journal.pone.0341774)
Supplement: S1 Additional Analysis — (DOCX) [file pone.0341774.s005.docx]

**Additional Analysis**

Item-level H value analysis

We subsampled (100 times) the 51-65 group to have an equal sample size as the other two groups (N=24). Permutation-based ANOVA analyses (N=1000 randomizations) on H values with subsampled data sets indicated that the main effect of age group was significant (all p<0.010). The mean consistency between the subsampled and full sample H values (assessed as Spearman’s rho of H values) for the 51-65 group was 0.96 (range = 0.94-0.97). Subsample analysis confirms that there was a minimal impact of sample size differences on H value in our cohort.
